# An Evaluation of International Standards for Neurological Classification of Spinal Cord Injury (ISNCSCI) Performance Within the Canadian SCI Network

**DOI:** 10.46292/sci25-00011

**Published:** 2025-08-22

**Authors:** Heather A. Hong, Jessica Parsons, Jijie Xu, Kristen Walden, Nader Fallah, Christiana L. Cheng, Najmedden Attabib, Sean D. Christie, B. Catharine Craven, Michael G. Fehlings, Daryl R. Fourney, Chester Ho, Lisa Julien, Brian K. Kwon, Gary A. Linassi, Adalberto Loyola-Sanchez, Saranjan Moganathas, Jerome Paquet, Vidya Sreenivasan, Jean-Marc Mac-Thiong, Andrea Townson, Eve C. Tsai, Jennifer Urquhart, Alexander Whelan, Vanessa K. Noonan, ISNCSCI Working Group, G. Adetunji, H. Ahn, A. Alludino, T. Anthony, M. Anton, N. Attabib, M. Boulet, L. Brisbois, S. Casha, M. Chabot, C. Cheng, S. Christie, S. Cole, I. Cote, B. C. Craven, P. Dhaliwal, W. D'Mello, J. Duley, M. Dvorak, J. Earle, N. Fallah, M. G. Fehlings, D. Fourney, C. Ho, H. A. Hong, L. Julien, H. Kainth, E. Khan, S. D. Kodamanchali, B. Kwon, J. Larouche, G. Linassi, C. Lownie, A. Loyola-Sanchez, S. Macleod, J.-M. Mac-Thiong, C. McGillivray, S. McVeigh, S. Middelton, S. Moganathas, K. Montgomery, V. K. Noonan, C. O'Connell, Y. Okuma, J. Paquet, J. Parsons, Y. Petrenko, C. Rahn, J. Reader, E. Richardson, L. Ritchie, G. Snedden, V. Sreenivasan, K. Stewart, F. R. Sydney, A. Townson, E. C. Tsai, J. Urquhart, J. Van Camp, K. Walden, A. Whelan, J. Xu

**Affiliations:** 1 Praxis Spinal Cord Institute, Vancouver, British Columbia, Canada; 2 Department of Medicine, University of British Columbia, Vancouver, British Columbia, Canada; 3 Division of Neurosurgery, Horizon Health Network, Dalhousie University, Saint John, New Brunswick, Canada; 4 Department of Surgery (Neurosurgery), Dalhousie University, Nova Scotia Health, Halifax, Nova Scotia, Canada; 5 KITE Research Institute, University Health Network. Toronto, Ontario, Canada; 6 Department of Medicine, Temerty Faculty of Medicine, University of Toronto, Toronto, Ontario, Canada; 7 Department of Kinesiology and Health Science, University of Waterloo, Waterloo, Ontario, Canada; 8 Rehabilitation Sciences Institute, Temerty Faculty of Medicine, University of Toronto, Toronto, Ontario, Canada; 9 Institute of Health Policy Management and Evaluation, University of Toronto, Toronto, Ontario, Canada; 10 Division of Neurosurgery and Spine Program, Department of Surgery, University of Toronto, Toronto, Ontario, Canada; 11 Division of Neurosurgery, Krembil Neuroscience Centre, Toronto Western Hospital, University Health Network, Toronto, Ontario, Canada; 12 Department of Surgery, University of Saskatchewan, Saskatoon, Saskatchewan, Canada; 13 Division of Physical Medicine and Rehabilitation, Department of Medicine, University of Alberta, Edmonton, Alberta, Canada; 14 NSHA Queen Elizabeth II Health Sciences Centre, Halifax, Nova Scotia, Canada; 15 Department of Orthopaedics, University of British Columbia, Vancouver, British Columbia, Canada; 16 International Collaboration on Repair Discoveries (ICORD), Faculty of Medicine, University of British Columbia, Vancouver, British Columbia, Canada; 17 Department of Physical Medicine and Rehabilitation, University of Saskatchewan, Saskatoon, Saskatchewan, Canada; 18 Division of Physical Medicine and Rehabilitation, University of Alberta, Edmonton, Canada; 19 Department of Clinical Neurosciences, Alberta Health Services, Edmonton, Canada; 20 Sunnybrook Hospital, Toronto, Ontario, Canada; 21 Centre de Recherche CHU de Quebec, CHU de Quebec-Universite Laval, Quebec, Quebec, Canada; 22 University of Ottawa, Faculty of Medicine, Ottawa, Ontario, Canada; 23 Faculty of Medicine, Université de Montréal, Centre-ville, Montréal, Québec, Canada; 24 Research Center, CIUSSS Nord-de-l’Île-de-Montréal, Hôpital du Sacré-Cœur de Montréal, Montréal, Québec, Canada; 25 Hôpital du Sacré-Cœur de Montréal, Montréal, Québec, Canada; 26 University of British Columbia, Vancouver, British Columbia, Canada; 27 Division of Surgery, Department of Surgery, Faculty of Medicine, University of Ottawa, Ottawa, Ontario, Canada; 28 Neuroscience Program, Ottawa Hospital Research Institute, Ottawa, Ontario, Canada; 29 London Health Sciences Centre Combined Neurosurgical and Orthopaedic Spine Program, Schulich School of Medicine, Western University, London, Ontario, Canada; 30 Department of Medicine (Physical Medicine and Rehabilitation), Dalhousie University, Halifax, Nova Scotia, Canada; 31 International Collaboration on Repair Discoveries (ICORD), University of British Columbia, Vancouver, British Columbia, Canada

**Keywords:** classification accuracy, clinical examination, completion, International Standards for Neurological Classification of Spinal Cord Injury (ISNCSCI), performance, spinal cord injury

## Abstract

**Objectives:**

To describe the performance of the International Standards for Neurological Classification of Spinal Cord Injury (ISNCSCI) examination in individuals with traumatic spinal cord injury (TSCI) and nontraumatic spinal cord injury (NTSCI) across Canadian acute and rehabilitation facilities, evaluating timing, completeness, and classification accuracy.

**Methods:**

Using the Rick Hansen Spinal Cord Injury Registry (2015-2022), participants were analyzed across 6 cohorts: (A) TSCI-acute-admission (*n* = 4461), (B) TSCI-acute-discharge (*n* = 972), (C) TSCI-rehabilitation-admission (*n* = 2673), (D) TSCI-rehabilitation-discharge *(n* = 2316), (E) NTSCI-rehabilitation-admission (*n* = 728), and (F) NTSCI-rehabilitation-discharge *(n* = 619). ISNCSCI data included performed (yes/no), timing (≤72 hours, ≤7 days, and >7 days of admission/discharge), completeness, missing items, and worksheet used (yes/no). Classification accuracy between the clinician-determined and algorithm-generated ASIA Impairment Scale and neurological level of injury classification was evaluated. Descriptive and bivariate statistics were used to analyze cohorts.

**Results:**

Overall, 70% of participants had at least one examination performed, with 76% performed ≤72 hours, 91% ≤7 days, and 9% >7 days. However, 45% were partially complete, primarily missing sensory scores and rectal components ≤7 days. Comparison of TSCI and NTSCI during rehabilitation showed that NTSCI cohorts had significantly more exams at admission and fewer at discharge, with more complete exams. Moreover, age at injury, injury type, mechanism, severity, length of stay, and pain influenced examination performance.

**Conclusion:**

This study highlights the need for greater consistency in ISNCSCI examination performance and identifies patient-level barriers to completion. Determining the most effective standardized approach for ISNCSCI use across SCI care, addressing modifiable human/organizational factors, and ensuring comprehensive clinical training will improve the quality of this assessment.

## Introduction

The International Standards for Neurological Classification of Spinal Cord Injury (ISNCSCI) examination, developed by the American Spinal Injury Association (ASIA), is the gold standard for assessing the level and severity of spinal cord injury (SCI).^1^-^5^ The bedside assessment of motor and sensory function provides scores to classify the neurological level of injury (NLI) and ASIA Impairment Scale (AIS) based on ISNCSCI classification rules.^4^ Accurate and standardized examinations are essential for diagnosis, prognosis, treatment planning, monitoring neurological changes, and defining clinical trial eligibility.^6^-^8^

The Rick Hansen Spinal Cord Injury Registry (RHSCIR), established in 2004, is a prospective registry tracking individuals with traumatic spinal cord injury (TSCI) across Canadian acute and rehabilitation facilities.^9^ Since 2006, RHSCIR has worked to improve clinical ISNCSCI use through a range of implementation efforts, including hands-on training, access to the online ASIA InSTeP course (https://asia-spinalinjury.org/learning/), development of a computerized classification algorithm,^10^ and the toolkit for SCI neurology assessment.^11^ In 2020, RHSCIR expanded to include individuals with nontraumatic spinal cord injury (NTSCI) attending inpatient rehabilitation. Accurate ISNCSCI exams are essential to the RHSCIR dataset. This study evaluates the impact of ISNCSCI implementation by assessing the network's performance in conducting and completing exams across RHSCIR facilities.

Examining clinical practices and patient factors that impact ISNCSCI performance across care settings can highlight whether ISNCSCI variations occur based on facility type (acute vs. rehabilitation), injury type (TSCI vs. NTSCI), or other patient factors. This study aims to (1) describe ISNCSCI examination performance (yes/no), timing (≤72 hours, ≤7 days, and >7 days of admission/discharge), completeness (partial: missing one/more ISNCSCI items; full: having all items present), and worksheet use (yes/no); (2) assess classification accuracy; and (3) identify factors associated with examination performance in individuals with TSCI and NTSCI in acute and rehabilitation facilities across Canada.

## Methods

### Database

This retrospective study used the RHSCIR 2015-2022 database. All RHSCIR sites obtained approval from the local research ethics board before enrolling participants.^9^

### Study cohorts

All participants admitted to an acute or rehabilitation facility were included and grouped into relevant cohorts based on SCI type (TSCI/NTSCI), care setting (acute/rehabilitation), and examination timing (admission/discharge). Cohorts were (A) TSCI-acute-admission, (B) TSCI-acute-discharge (i.e., not attending RHSCIR facility inpatient rehabilitation), (C) TSCI-rehabilitation-admission, (D) TSCI-rehabilitation-discharge, (E) NTSCI-rehabilitation-admission, and (F) NTSCI-rehabilitation-discharge. Due to their distinct causes, severity, cohort characteristics (e.g., age, complications), and neurological prognoses,^12^-^15^ as well as the recent validation of ISNCSCI use in individual with NTSCI,^5^ TSCI and NTSCI were analyzed separately.

### Study variables

Variables included demographics (age at injury, sex, ethnicity, language), injury factors (TSCI mechanism, Glasgow Coma Scale [GCS], Injury Severity Score [ISS], spine surgery, airway interventions, NTSCI etiology, and NTSCI onset timeframe), and care factors (length of stay [LOS] for acute and rehabilitation, and discharge destination from acute and rehabilitation).

ISNCSCI examination data were categorized as follows:

- *Performed*: “Yes” (some bedside examination data, including motor, sensory, voluntary anal contraction [VAC], deep anal pressure [DAP], and a date; the most complete examination was used if multiple examinations were performed), “No” (absent bedside examination data or only AIS and NLI classification), and “Missing-Date” (bedside examination missing date). Data also included reasons for not performing the bedside examination, such as traumatic brain injury (TBI), mental illness, death, discharge, nonconsent, or other factors.
- *Timing*: Stratified by examination performance timing ≤72 hours, ≤7 days, or >7 days post admission or prior to discharge. For cohort C, ±7 days was used, as some facilities complete the rehabilitation admission ISNCSCI via consultation before acute care discharge.
- *Completeness*: “Partial” (missing items) and “Full” (complete examination, including all motor and sensory scores, VAC, DAP, AIS, and NLI). The Zone of Partial Preservation was not required.
- *Worksheet used*: “Yes” (medical record data recorded on the standardized ISNCSCI work sheet [https://asia-spinalinjury.org/wp-content/uploads/2023/12/ASIA-ISCOS-Worksheet-Sides-12_12_4_2023.pdf]^16^) and “No” (medical record data extracted from a non-ISNCSCI worksheet source, e.g., free-text clinical notes).
- *Classification accuracy*: Assessed by entering the clinician's bedside examination scores into the ISNCSCI algorithm (https://www.isncscialgorithm.com/) 10 to generate AIS and NLI classifications. For full examinations, an AIS and NLI match occurred when the clinician's classifications matched the algorithm's classifications. For partial examinations, where the algorithm-generated AIS or NLI was a range of possible values, a match was when the clinician's classification fit within that range.

### Statistical analyses

Descriptive statistics (median, interquartile range [IQR], counts, percentages) were calculated for each cohort. Bivariate analysis identified factors linked to examination performance: “No” versus “Yes” examination (partial/full) ≤7 days. Student *t* tests, Wilcoxon rank sum tests, chi-square tests, and Fisher's exact test assessed group differences (depending on data distribution). Statistical analyses were conducted using SAS 9.4 and SAS Stats 15.1 (SAS Institute Inc., Cary, NC), with a *P* value <.05 considered significant.

## Results

### Performance, timing, and completion

**Figure 1.**
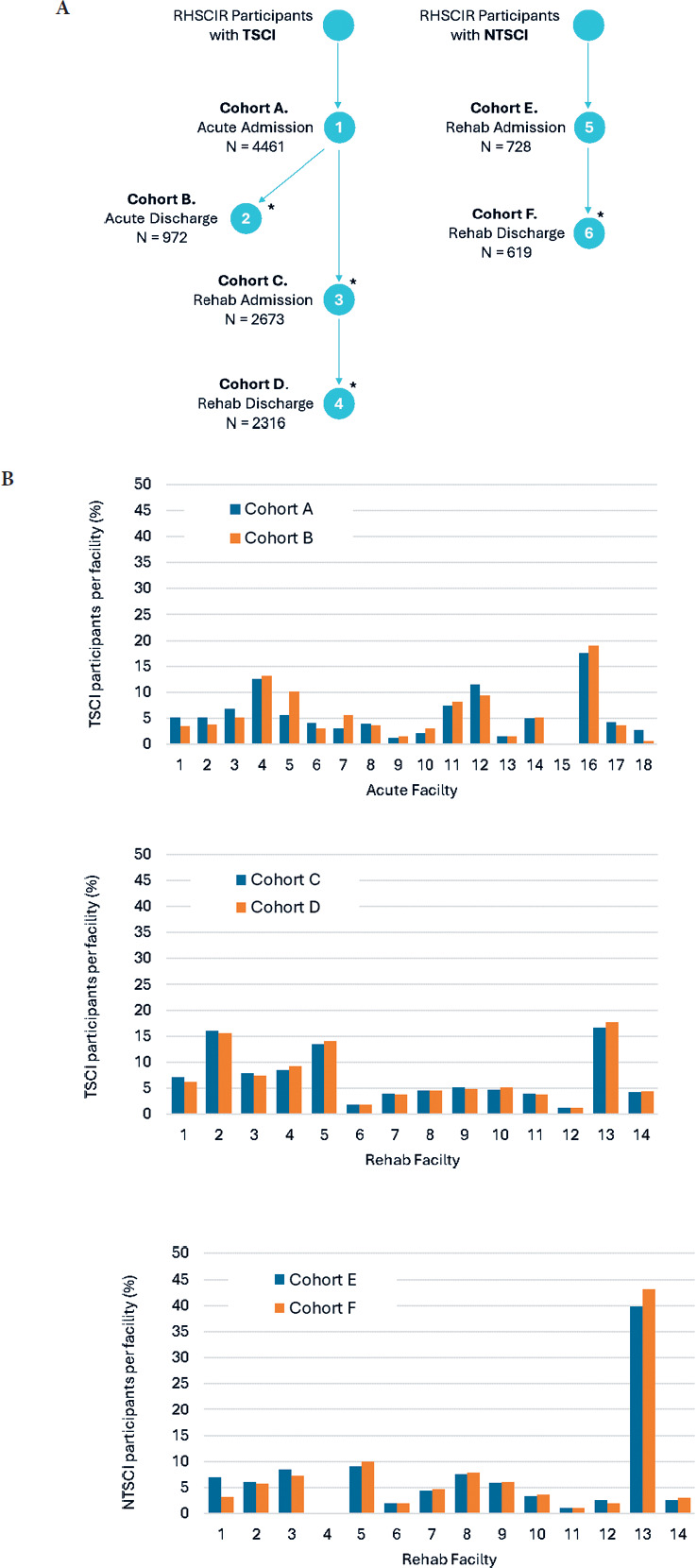
(**A**) Overview of the 6 ISNCSCI cohorts and sample size from the Rick Hansen Spinal Cord Injury Registry (RHSCIR) 2015-2022 database. Cohort A (TSCI-acute-admission), B (TSCI-acute-discharge, i.e., not attending RHSCIR facility inpatient rehabilitation), C (TSCI-rehabilitation-admission), D (TSCI-rehabilitation-discharge), E (NTSCI-rehabilitation-admission), and F (NTSCI-rehabilitation-discharge). *Note: Participants from cohort A (*n* = 4461) who were not included in cohort B (*n* = 972) or cohort C (*n* = 2673) were still acute care facility inpatients when data was abstracted (*n* = 830) or had missing/other discharge destinations (*n* = 24). Likewise, participants in cohort C (*n* = 2673) absent from cohort D (*n* = 2316) remained rehabilitation facility inpatients (*n* = 324) or had missing/other discharge destinations (*n* = 33), and participants in cohort E (*n* = 728) missing from cohort F (*n* = 619) remained rehabilitation facility inpatients (*n* = 97) or had missing/other discharge destinations (*n* = 12). (**B**) Comparison of participant frequencies in participants with TSCI and NTSCI across acute and rehabilitation facilities. AIS = ASIA Impairment Scale; DAP = deep anal pressure; ISNCSCI = International Standards for Neurological Classification of Spinal Cord Injury; NLI = neurological level of injury; NTSCI = nontraumatic spinal cord injury; rehab = rehabilitation; TSCI = traumatic spinal cord injury; VAC = voluntary anal contraction.

Cohorts A-D included 4461, 972, 2673, and 2316 participants with TSCI, while cohorts E and F included 728 and 619 participants with NTSCI (**Figures 1A**). Among the 18 acute care and 14 rehabilitation facilities, the highest proportion of participants for cohorts A (TSCI-acute-admission) and B (TSCI-acute-discharge) were from acute facility 16 (**Figure 1B**). For cohorts C (TSCI-rehabilitation-admission) and D (TSCI-rehabilitation-discharge), the largest proportion of participants was from rehabilitation facilities 2 (16%) and 13 (17%). For cohorts E (NTSCI-rehabilitation-admission) and F (NTSCI-rehabilitation-discharge), facility 13 had the highest proportion of participants (40%).

Overall, 70% of participants had an examination performed, 26% had none, and 4% were missing the date (**Table 1**). Among participants with an examination, 76% were completed ≤72 hours, 91% were completed ≤7 days, and 9% were completed >7 days from admission or discharge. Full completion was higher in examinations completed >7 days (61%) as compared to examinations completed ≤7 days (55%) or ≤72 hours (54%).

**Table 1.** ISNCSCI examination across cohorts A to F using the RHSCIR 2015-2022 database

ISNCSCI examination	Cohort A TSCI-acute-admission	Cohort B TSCI-acute-discharge	*P* value	Cohort C TSCI-rehab-admission	Cohort D TSCI-rehab-discharge	*P* value	Cohort E NTSCI-rehab-admission	Cohort F NTSCI-rehab-discharge	*P* value	Average^e^
***Performed, n (%)***										
Participants	4461	972		2673	2316		728	619		1962
Yes^a^	3607 (80.9)	348 (35.8)	**<.0001**	2112 (79.0)	1246 (53.8)	**<.0001**	647 (88.9)	225 (36.3)	**<.0001**	1364 (69.5)
No^b^	588 (13.2)	600 (61.7)		444 (16.6)	989 (42.7)		60 (8.2)	379 (61.2)		510 (26.0)
Missing date	266 (6.0)	24 (2.5)		117 (4.4)	81 (3.5)		21 (2.9)	15 (2.4)		87 (4.5)
***Timing, n (%)***										
Yes exam	3607	348		2112	1246		647	225		1364
Exam <72 h^c^	2998 (83.1)	237 (68.1)	**<.0001**	1657 (78.5)	736 (59.1)	**<.0001**	464 (71.7)	162 (72.0)	.9383	1042 (76.4)
Exam <7 d	3406 (94.4)	291 (83.6)	**<.0001**	1951 (92.4)	976 (78.3)	**<.0001**	602 (93.0)	197 (87.6)	**.0104**	1237 (90.7)
Exam >7 d	201 (5.6)	57 (16.4)		161 (7.6)	270 (21.7)		45 (7.0)	28 (12.4)		127 (9.3)
***Completion, n (%)***										
Exam <72 h	2998	237		1657	736		464	162		1042
Partial^d^	1653 (55.1)	136 (57.4)	.5028	607 (36.6)	323 (43.9)	**.0008**	117 (25.2)	68 (42.0)	**.0001**	484 (46.4)
Full^d^	1345 (44.9)	101 (42.6)		1050 (63.4)	413 (56.1)		347 (74.8)	94 (58.0)		558 (53.6)
Exam <7 d	3406	291		1951	976		602	197		1237
Partial	1814 (53.3)	163 (56.0)	.3658	713 (36.5)	429 (44.0)	**.0001**	151 (25.1)	79 (40.1)	**.0001**	558 (45.1)
Full	1592 (46.7)	128 (44.0)		1238 (63.5)	547 (56.0)		451 (74.9)	118 (59.9)		679 (54.9)
Exam >7 d	201	57		161	270		45	28		127
Partial	99 (49.3)	22 (38.6)	.1546	54 (33.5)	114 (42.2)	.0738	7 (15.6)	4 (14.3)	.8848	50 (39.4)
Full	102 (50.7)	35 (61.4)		107 (66.5)	156 (57.8)		38 (84.4)	24 (85.7)		77 (60.6)
***Absent ISNCSCI items in partial examinations <7 d, n (%)***										
Exam	1814	163		713	429		151	79		558
Any motor score	212 (11.7)	25 (15.3)	.1692	39 (5.5)	29 (6.8)	.3723	6 (4.0)	4 (5.1)	.7005	53 (9.4)
Any LT sensory score	972 (53.6)	88 (54.0)	.9244	395 (55.4)	269 (62.7)	**.0154**	22 (14.6)	22 (27.8)	**.0151**	295 (52.8)
Any PP sensory score	1047 (57.7)	91 (55.8)	.6406	383 (53.7)	268 (62.5)	**.0038**	24 (15.9)	22 (27.8)	**.0314**	306 (54.8)
VAC	706 (38.9)	92 (56.4)	**<.0001**	341 (47.8)	248 (57.8)	**.0011**	84 (55.6)	59 (74.7)	**.0047**	255 (45.7)
DAP	841 (46.4)	105 (64.4)	**<.0001**	337 (47.3)	245 (57.1)	**.0013**	82 (54.3)	57 (72.2)	**.0086**	278 (49.8)
Neurological level, sensory	741 (40.8)	64 (39.3)	.6929	174 (24.4)	93 (21.7)	.2919	18 (11.9)	12 (15.2)	.4844	184 (32.9)
Neurological level, motor	706 (38.9)	49 (30.1)	**.0258**	163 (22.9)	75 (17.5)	**.0302**	20 (13.2)	12 (15.2)	.6855	171 (30.6)
NLI	570 (31.4)	45 (27.6)	.3135	114 (16.0)	52 (12.1)	.0725	13 (8.6)	8 (10.1)	.7034	134 (23.9)
Complete/ Incomplete	356 (19.6)	56 (34.4)	**<.0001**	71 (10.0)	58 (13.5)	.0656	24 (15.9)	18 (22.8)	.1991	97 (17.4)
AIS	367 (20.2)	26 (16.0)	.1896	59 (8.3)	46 (10.7)	.1656	17 (11.3)	8 (10.1)	.7928	87 (15.6)

Note: The *P* values are calculated for each individual variable (e.g., timing <72 hours) or variable category (e.g., exam timing <7 days vs. >7 days) to assess differences between groups (Cohort A vs. Cohort B, Cohort C vs. Cohort D, and Cohort E vs. Cohort F).

AIS = ASIA Impairment Scale; d = days; DAP = deep anal pressure; h = hours; ISNCSCI = International Standards for Neurological Classification of Spinal Cord Injury; LT = light touch; NLI = neurological level of injury; NTSCI = nontraumatic spinal cord injury; PP = pinprick; rehab = rehabilitation; RHSCIR = Rick Hansen Spinal Cord Injury Registry; TSCI = traumatic spinal cord injury; VAC = voluntary anal contraction.

a Yes examination = participants with bedside examination data, including motor, sensory, VAC, or DAP scores and a date; the most complete examination was used for those with multiple examinations.

b No examination = participants with no bedside examination data or only an AIS and NLI classification available.

c There is an overlap between the number of examinations performed <72 hours and <7 days of admission/discharge depending on the cohort.

d Partial examinations have one/more missing ISNCSCI items, and full examinations have all items present (i.e., motor and sensory scores, VAC, DAP, AIS, and NLI).

e Average = sum of cohorts A to F divided by 6 and rounded up.

Examination performance varied across cohorts (**Table 1**), with more examinations performed at admission compared to discharge (cohorts A vs. B, C vs. D, and E vs. F; all *P*s < .001). Moreover, rehabilitation admission examinations were more complete than discharge examinations both for those performed ≤72 hours (cohorts C vs. D: *P* = .0008; cohorts E vs. F; *P* = .0001) and for those performed ≤7 days (cohorts C vs. D; cohorts E vs F; both *P*s = .0001). Acknowledging the higher proportion and the greater completeness of examinations performed ≤7 days, all further analysis focused on these examinations.

### Missing items in partial examinations

**Figure 2.**
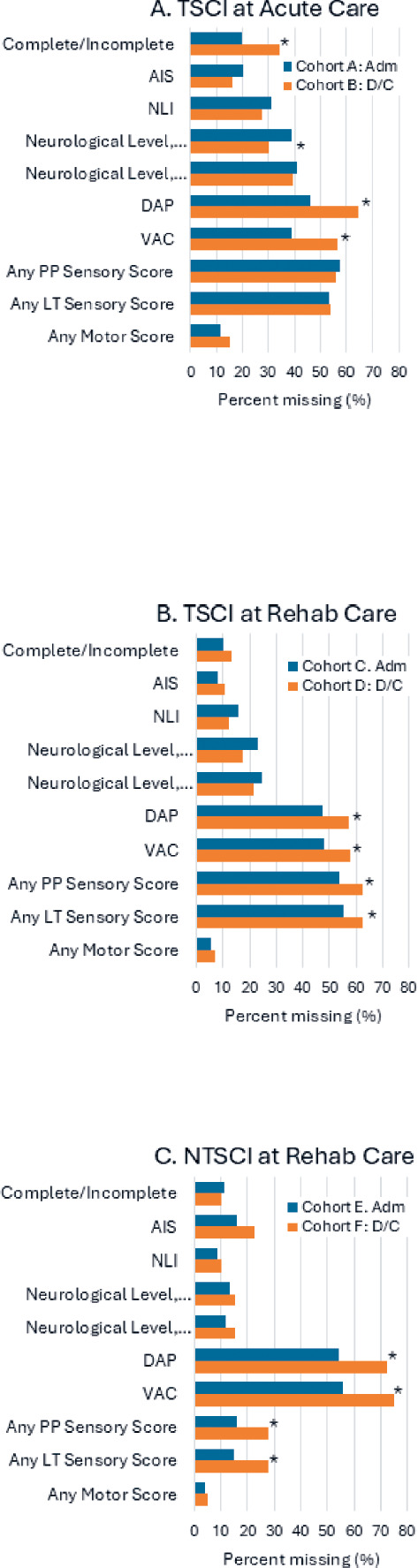
Comparison of missing ISNCSCI items in partial examinations performed within 7 days across: (**A**) TSCI at acute care: cohort A (TSCI-acute-admission) vs. cohort B (TSCI-acute-discharge); (**B**) TSCI at rehabilitation care: cohort C (TSCI-rehabilitation-admission) vs. cohort D (TSCI-rehabilitation-discharge); and (**C**) NTSCI at rehabilitation care: cohort E (NTSCI-rehabilitation-admission) vs. cohort F (NTSCI-rehabilitation-discharge). *Indicates a statistically significant difference between admission and discharge within each care setting. A partial examination is missing one/more elements. Adm = admission; AIS = ASIA Impairment Scale; DAP = deep anal pressure; D/C = discharge; ISNCSCI = International Standards for Neurological Classification of Spinal Cord Injury; LT = light touch; NLI = neurological level of injury; NTSCI = nontraumatic spinal cord injury; PP = pinprick; TSCI = traumatic spinal cord injury; VAC = voluntary anal contraction.

In both TSCI and NTSCI cohorts, across both acute and rehabilitation, the most frequently absent items in partial examinations performed within 7 days of admission/discharge were sensory scores (pinprick [PP] 55%, light touch [LT] 53%) and rectal items (DAP 50%, VAC 46%; **Figure 2**). In contrast, motor scores had the lowest rate of absence (9%). Moreover, DAP and VAC had significantly higher absence at discharge compared to admission.

### Worksheet use and classification accuracy

**Figure 3.**
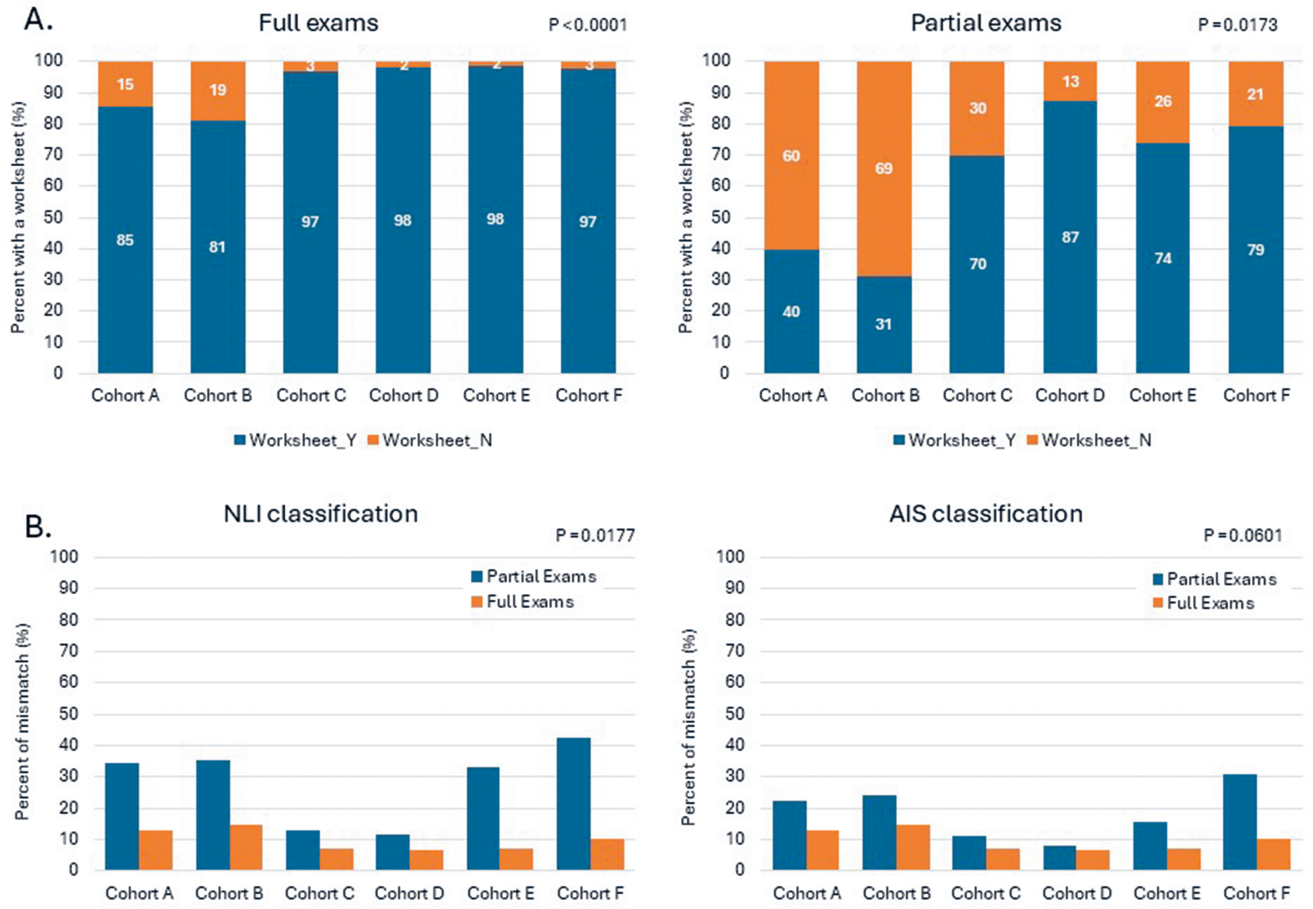
(**A**) Comparison of the percentage of full examinations with a worksheet and partial examinations with a worksheet across cohorts A to F. (**B**) Comparison of classification accuracy (the percent of mismatch between the clinician-determined and algorithm-generated AIS and NLI classification) in partial and full examinations across cohorts A to F. AIS = ASIA Impairment Scale; Cohorts = A: TSCI-acute-admission; B: TSCI-acute-discharge (i.e., not attending RHSCIR facility inpatient rehabilitation); C: TSCI-rehabilitation-admission; D: TSCI-rehabilitation-discharge, E: NTSCI-rehabilitation-admission; F: NTSCI-rehabilitation-discharge; ISNCSCI = International Standards for Neurological Classification of Spinal Cord Injury; NLI = neurological level of injury; NTSCI = nontraumatic spinal cord injury; rehab = rehabilitation; TSCI = traumatic spinal cord injury.

Worksheets were used in 93% of full examinations and 64% of partial examinations (**Figure 3A**). A comparison of NLI and AIS classification accuracy showed that full examinations had a significantly lower mismatch rate compared to partial examinations (**Figure 3B**). Not surprisingly, partial examinations had a significantly higher mismatch rate for the NLI classification (28%) than the AIS classification (19%).

### ISNCSCI performance comparison: TSCI vs. NTSCI

**Figure 4.**
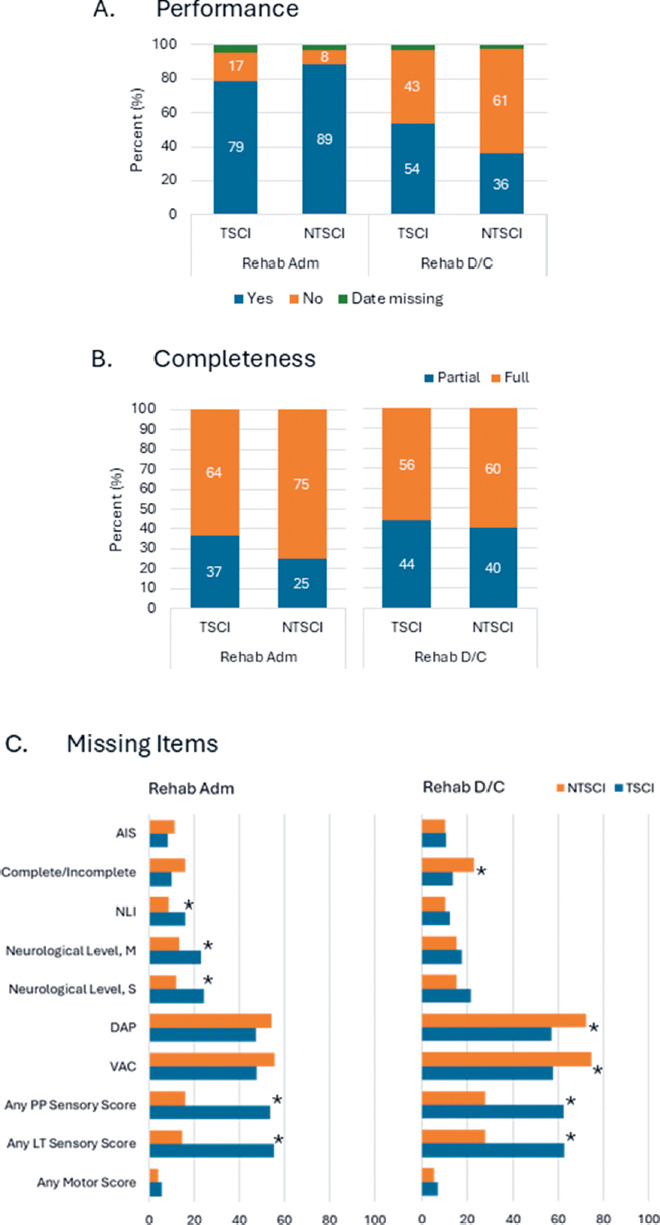
(**A**) Comparison of ISNCSCI examination performance (yes, no, and date missing) in individuals with TSCI and NTSCI at rehabilitation. (**B**) Comparison of the percentage of complete ISNCSCI examinations conducted in individuals with TSCI and NTSCI within 7 days of rehabilitation. (**C**) Comparison of missing items in partial ISNCSCI examinations between individuals with TSCI and NTSCI within 7 days of rehabilitation. A partial examination is missing one/more elements. *Indicates a significant difference in the number of missing items between TSCI and NTSCI groups. Adm = admission; AIS = ASIA Impairment Scale; DAP = deep anal pressure; D/C = discharge; ISNCSCI = International Standards for Neurological Classification of Spinal Cord Injury; LT = light touch; M = motor; NLI = neurological level of injury; NTSCI = nontraumatic spinal cord injury; PP = pinprick; S = sensory; TSCI = traumatic spinal cord injury; VAC = voluntary anal contraction.

A comparison of ISNCSCI performance showed significantly more exams performed at rehabilitation admission in the NTSCI cohort, but fewer at discharge, compared to the TSCI cohort (**Figure 4A**). Additionally, the TSCI cohort had a significantly higher number of partial exams at rehabilitation admission than the NTSCI cohort (**Figure 4B**). Among these partial exams, sensory scores (LT and PP) were significantly more often absent in the TSCI cohort compared to the NTSCI cohort, both at admission and discharge (**Figure 4C**). In contrast, anal components (VAC and DAP) were significantly absent in the NTSCI cohort compared to the TSCI cohort.

### Factors associated with ISNCSCI performance

For cohort A, the median age at injury for participants with TSCI at acute admission was 53 years, 76% were male, 51% reported falling as the primary mechanism of injury, and the median acute care stay was 21 days. Bivariate analysis found that ethnicity, language, injury mechanism, GCS, ISS, having spine surgery, presence of an oral/nasal endotracheal tube, and tracheostomy were significantly associated with examinations performed (**Table 2**). In cohort B, these factors, along with age at injury, acute LOS, and discharge destination, were also significantly linked to examination performance at acute discharge.

**Table 2.** Comparison of factors associated with ISNCSCI examination performance within 7 days for cohort A (TSCI-acute-admission) and cohort B (TSCI-acute-discharge)

Variables		Cohort A			Cohort B	
	No exam (*n*=588)	Yes exam <7 d (*n*=3406)	*P* value	No exam (*n*=600)	Yes exam <7 d (*n*=291)	*P* value
***Demographic factors***						
Age at injury, years, median (IQR)	54 (34)	55 (32.0)	.9824	62 (32)	52 (31)	**<.0001**
Sex at birth, male, *n (%)*	431 (73.3)	2610 (76.6)	.0802	446 (74.3)	222 (76.3)	.5275
Ethnicity, *n (%)*						
Caucasian	135 (77.1)	1317 (83.5)	**.0016**	84 (84.0)	81 (80.2)	.2507
Non-Caucasian	40 (22.8)	260 (16.5)		16 (16.0)	20 (19.8)	
Language, *n (%)*						
English	141 (78.8)	1179 (74.3)	**.0087**	84 (81.5)	71 (69.6)	**.0323**
Non-English/ other/unknown	38 (21.2)	407 (25.7)		19 (18.5)	31 (30.4)	
***Injury factors***						
Mechanism of injury, *n (%)*						
Sports	59 (10.0)	498 (14.6)		70 (11.7)	66 (22.7)	
Assault	23 (3.9)	138 (4.0)		13 (2.2)	5 (1.7)	
Transport	189 (32.1)	835 (24.5)	**.0002**	146 (24.3)	67 (23.0)	**.0001**
Fall	288 (49.0)	1770 (52.0)		344 (57.3)	138 (47.4)	
Other	19 (3.2)	139 (4.1)		20 (3.3)	15 (5.1)	
Unknown	10 (1.7)	26 (0.8)		7 (1.2)	0 (0.0)	
GCS severity groups, *n (%)*						
Severe, GCS 3-8	86 (14.6)	54 (1.6)		60 (10)	6 (2.1)	
Moderate, GCS 9-12	27 (4.6)	82 (2.4)	**<.0001**	28 (4.7)	4 (1.4)	**<.0001**
Mild, GCS 13-15	308 (52.4)	2769 (81.3)		368 (61.3)	247 (84.9)	
Missing	167 (28.4)	501 (14.7)		144 (24.0)	34 (11.7)	
ISS groups, *n (%)*						
<25	236 (40.1)	1768 (51.9)		314 (52.3)	163 (56.0)	
*>25*	182 (30.9)	687 (20.2)	**<.0001**	130 (21.7)	25 (8.6)	**<.0001**
Unknown	170 (28.9)	951 (27.9)		156 (26.0)	103 (35.4)	
Spine surgery (yes), *n (%)*	439 (75.0)	2906 (85.3)	**<.0001**	376 (62.7)	225 (77.3)	**<.0001**
Oral/Nasal endotracheal tube >24 h (yes), *n (%)*	272 (46.4)	843 (24.7)	**<.0001**	219 (36.6)	33 (11.3)	**<.0001**
Tracheostomy <7 d of admission (yes), *n (%)*	108 (18.7)	383 (11.3)	**<.0001**	73 (12.2)	8 (2.7)	**<.0001**
***Acute factors***						
Acute LOS (days), median (IQR)	24 (38)	21 (24)	.1137	12 (23)	9.5 (9)	**<.0001**
Discharge destination from acute, *n (%)*						
Hospital	369 (62.8)	2681 (78.7)		-	-	
Private residence	70 (11.9)	463 (13.6)	**<.0001**	272 (45.3)	242 (83.2)	**<.0001**
Morgue	110 (18.7)	169 (5.0)		251 (41.8)	29 (10.0)	
Unknown/Other^*^	39 (6.6)	93 (2.7)		77 (12.8)	20 (6.8)	

*Note:* For cohort A, ISNCSCI examinations conducted within 7 days of admission were used. For cohort B, ISNCSCI examinations conducted within 7 days prior to discharge were used. The *P* values are calculated for each individual variable (e.g., age at injury) or variable category (e.g., Ethnicity: Caucasian/Non-Caucasian) within the groups (i.e., No exam vs. Yes exam).

* Other discharge destinations include nursing home/long-term care in a hospital setting, assisted living residence, group living arrangement, homelessness, or correctional institute. d = days; GCS = Glasgow Coma Scale; h = hours; ISNCSCI = International Standards for Neurological Classification of Spinal Cord Injury; ISS = Injury Severity Score; IQR = interquartile range; LOS = length of stay; TSCI = traumatic spinal cord injury.

In cohort C, the median age at injury for participants with TSCI attending rehabilitation was 51, 78% were male, 46% reported falls as the mechanism of injury, and the median rehabilitation stay was 74 days. Language and pain presence during rehabilitation were significantly associated with examination completion (**Table 3**). In cohort D, the mechanism of injury and rehabilitation LOS were significantly linked to examination performance at rehabilitation discharge.

**Table 3.** Comparison of factors associated with ISNCSCI examination performance within 7 days for Cohort C (TSCI-rehabilitation-admission) and Cohort D (TSCI-rehabilitation-discharge)

Variable	Cohort C			Cohort D		*P* value
	No exam (*n* = 444)	Yes exam ±7 d (*n* = 1951)	*P* value	No exam (*n* = 989)	Yes exam ≤7 d (*n* = 976)	
***Demographic factors***						
Age at injury (years), median (IQR)	49 (33)	51 (32)	.2279	51 (34)	49 (31)	.824
Sex (male), *n* (%)	342 (77.0)	1535 (78.7)	.4458	760 (76.8)	768 (78.7)	.3259
Ethnicity, *n* (%)						
Caucasian	236 (85.2)	921 (84.2)	.2769	408 (83.4)	533 (83.4)	.3252
Non-Caucasian	41 (14.8)	173 (15.8)		81 (16.6)	106 (16.6)	
Language, *n* (%)						
English	164 (59.0)	820 (74.5)	**<.0001**	354 (72.2)	466 (72.1)	.092
Non-English, other, or unknown	114 (41.0)	280 (25.5)		136 (27.8)	180 (27.9)	
***Injury factors***						
Mechanism of injury, *n* (%)						
Sports	77 (17.3)	290 (14.9)	.5787	128 (12.9)	180 (18.4)	**.0003**
Assault	19 (4.3)	75 (3.8)		46 (4.6)	30 (3.1)	
Transport	123 (27.7)	575 (29.5)		305 (30.8)	260 (26.6)	
Fall	202 (45.5)	910 (46.6)		464 (46.9)	448 (45.9)	
Other	18 (4.0)	91 (4.7)		42 (4.2)	53 (5.4)	
Unknown	5 (1.1)	10 (0.5)		4 (0.4)	5 (0.5)	
GCS severity at facility, *n* (%)						
Severe, GCS 3-8	15 (3.4)	50 (2.6)	.6049	26 (2.6)	25 (2.6)	**.0349**
Moderate, GCS 9-12	14 (3.1)	53 (2.7)		32 (3.2)	23 (2.4)	
Mild, GCS 13-15	342 (77.0)	1553 (79.6)		760 (76.8)	800 (82.0)	
Missing	73 (16.4)	295 (15.1)		171 (17.3)	128 (13.1)	
ISS groups, *n* (%)						
≤25	221 (49.8)	943 (48.3)	.8589	473 (47.8)	490 (50.2)	**.0015**
>25	109 (24.5)	495 (25.4)		224 (22.6)	264 (27.0)	
Unknown	114 (25.7)	513 (26.3)		292 (29.5)	222 (22.7)	
***Rehabilitation factors***						
Acute LOS, days, median (IQR)	27 (31)	24 (26)	.0694	25 (28)	22 (22)	**.002**
Rehab LOS, days, median (IQR)	73 (75)	75 (61)	.4401	69 (64)	72 (56)	**.0147**
Delirium in rehab care, yes, *n* (%)	5 (1.4)	38 (2.5)	.2879	17 (2.3)	11 (1.4)	.1679
Any pain in rehab care, yes, *n* (%)	141 (93.4)	636 (86.5)	**.0491**	266 (87.5)	345 (89.1)	.7723
Any neuropathic in rehab care, yes, *n* (%)	46 (73.0)	285 (76.2)	.1229	153 (74.6)	138 (78.4)	.3696
Discharge destination from rehab, *n* (%)						
Private residence	322 (72.5)	1508 (77.3)	**.0037**	847 (85.6)	890 (91.2)	**.0012**
Hospital	69 (15.5)	221 (11.3)		3 (0.3)	0 (0.0)	
Unknown/Other^*^	51 (11.5)	217 (11.1)		133 (13.3)	86 (8.8)	

1 *Note:* For cohort C, ISNCSCI exams conducted within 7 days before or after admission were used, as some facilities perform the rehabilitation admission ISNCSCI via consultation prior to acute care discharge. For cohort D, ISNCSCI exams conducted within 7 days prior to discharge were used. The *P* values correspond to each individual variable (e.g., age at injury) or variable category (e.g., Ethnicity: Caucasian/Non-Caucasian) within the groups (i.e., No exam vs. Yes exam).

* Other discharge destinations include nursing home/long-term care in a hospital setting, assisted living residence, group living arrangement, hotel/motel, or correctional institute. d = days; GCS = Glasgow Coma Scale; ISNCSCI = International Standards for Neurological Classification of Spinal Cord Injury; ISS = Injury Severity Score; IQR = interquartile range;

In cohort E, the median age at injury for participants with NTSCI attending rehabilitation was 62, 48% reported degenerative disease as the injury etiology, and the median rehabilitation LOS was 55 days. Rehabilitation LOS and discharge destination were significantly associated with examination completion at rehabilitation admission, while the timeframe of NTSCI onset was associated with examination completion at rehabilitation discharge (**Table 4**).

**Table 4.** Comparison of factors associated with ISNCSCI examination performance within 7 days for Cohort E (NTSCI-rehabilitation-admission) and Cohort F (NTSCI-rehabilitation-discharge)

Variable	No exam (*n* = 60)	Cohort E Yes exam ≤7 d (*n* = 602)	*P* value	No exam (*n* = 379)	Cohort F	
					Yes exam ≤7 d (*n* = 197)	*P* value
***Demographic factors***						
Age at injury, years, median (IQR)	63 (16)	62 (20)	.7748	62 (19)	63 (20)	.6168
Sex at birth, male, *n* (%)	34 (56.7)	350 (58.1)	.8255	224 (59.1)	111 (56.3)	.5245
***Injury factors***						
Etiology of NTSCI, *n* (%)						
Congenital/genetic	1 (2.2)	6 (1.2)		5 (1.7)	2 (1.1)	
Degenerative disease	14 (31.1)	257 (49.3)		142 (47.2)	91 (48.9)	
Vascular disorder	2 (4.4)	29 (5.6)		18 (6.0)	12 (6.4)	
Tumor (benign/malignant)	10 (22.2)	95 (18.2)	.0634	57 (18.9)	36 (19.3)	.2655
Infection	8 (17.8)	64 (12.3)		31 (10.3)	27 (14.5)	
Other	3 (6.7)	49 (9.4)		34 (11.3)	11 (5.9)	
Inflammatory	7 (15.6)	21 (4.0)		14 (4.6)	7 (3.8)	
Timeframe of NTSCI onset						
Acute (≤1 day)	3 (5.0)	52 (8.6)		36 (9.5)	19 (9.6)	
Sub-acute (>1 day but ≤7 days)	9 (15.0)	97 (16.1)		41 (10.8)	40 (20.3)	
Prolonged (>7 days but ≤1 month)	11 (18.3)	75 (12.5)	.0887	44 (11.6)	32 (16.2)	**.0056**
Lengthy (>1 month)	22 (36.7)	292 (48.5)		193 (50.9)	80 (40.6)	
Unknown	15 (25.0)	86 (14.3)		65 (17.1)	26 (13.2)	
***Rehabilitation factors***						
Rehab LOS, days, median (IQR)	42 (39)	56 (44)	**.0252**	52 (47)	55 (36)	.6114
Any neuropathic in rehab, yes, *n* (%)	31 (64.6)	355 (67.0)	.6344	228 (71.2)	115 (63.2)	.1711
Discharge destination from rehab, *n* (%)						
Hospital	13 (21.7)	60 (10.0)		0 (0.0)	0 (0.0)	
Private residence	42 (70.0)	477 (79.2)	**.1921**	335 (88.4)	183 (92.9)	.1754
Unknown/Other^*^	5 (8.3)	65 (10.7)		44 (11.6)	14 (7.1)	

*Note:* For cohort E, ISNCSCI exams conducted within 7 days after admission were used. For cohort D, ISNCSCI exams conducted within 7 days prior to discharge were used. The *P* values are calculated for each individual variable (e.g., age at injury) or variable category (e.g., etiology types) within the groups (i.e., No exam vs. Yes exam).

* Other discharge destination includes nursing home/long-term care in a hospital setting. The *P* values are specific to each individual item or subgroup within groups (i.e., No exam vs Yes exam). d = days; ISNCSCI = International Standards for Neurological Classification of Spinal Cord Injury; IQR = interquartile range; LOS = length of stay; NTSCI = nontraumatic spinal cord injury.

### Reasons for no ISNCSCI examination

The most common reason for no ISNCSCI examination was unknown (e.g., not documented in the medical record) at 65%, followed by “other” (20%), discharge before examination (6%), death before examination (3%), concurrent TBI (3%), nonconsent (3%), and mental illness (0.7%) (**Table 5**). Mental illness was highest in cohorts A (TSCI-acute-admission) at 13%, and concurrent TBI was highest in cohort B (TSCI-acute-discharge) at 14%. Additionally, free-text responses under “other” showed that 67 of 176 (38%) participants in cohort A cited “sedation/intubation” as the reason for no examination at acute admission.

**Table 5.** Reasons for no ISNCSCI examination across cohorts A to F

	Cohort A TSCI-acute-admission	Cohort B TSCI-acute-discharge	Cohort C TSCI-rehab-admission	Cohort D TSCI-rehab-discharge	Cohort E NTSCI-rehab-admission	Cohort F NTSCI-rehab-discharge	Average^a^
*N*	789	657	605	1259	105	407	637
Mental illness	101 (12.8)	8 (1.2)	-	-	1 (1.0)	-	5 (0.7)
Nonconsented	20 (2.5)	3 (0.5)	3 (0.5)	2 (0.2)	-	-	17 (2.6)
Concurrent TBI	22 (2.8)	89 (13.5)	-	6 (0.5)	-	1 (0.2)	18 (2.9)
Died before exam	25 (3.2)	57 (8.7)	7 (1.2)	111 (8.8)	-	22 (5.0)	20 (3.1)
Discharged before exam	8 (1.0)	8 (1.2)	33 (5.5)	24 (1.9)	6 (5.7)	21 (5.2)	37 (5.8)
Other	178 (22.6)	60 (9.1)	186 (30.7)	269 (21.4)	20 (19.0)	35 (8.6)	125 (19.6)
Unknown	435 (55.1)	432 (65.8)	376 (62.1)	847 (67.3)	78 (74.3)	328 (80.6)	416 (65.3)

*Note:* NTSCI = nontraumatic spinal cord injury; rehab = rehabilitation; TBI = traumatic brain injury; TSCI = traumatic spinal cord injury.

a Average, sum of cohorts A to F divided by 6 and rounded up.

## Discussion

This retrospective study evaluated the ISNCSCI examination in participants with TSCI and NTSCI during the acute and rehabilitation phases of SCI care, with the goal of improving the clinical use of the ISNCSCI in the RHSCIR Network. ISNCSCI performance, timing, and completion rates varied significantly across the cohorts. On average, 70% of participants across all cohorts had at least one examination performed. Of those, 76% were performed within 72 hours, 91% within 7 days, and 9% after 7 days after admission or before discharge. Notably, more examinations were conducted at admission than discharge (comparing cohorts A vs. B, C vs. D, and E vs. F), suggesting that clinicians may prioritize the examination at admission to guide treatment and predict patient outcomes.

However, nearly half of the examinations were partially completed. In these partial examinations, AIS and NLI classification accuracy was also significantly lower compared to fully completed examinations, suggesting that incomplete examinations may lead to errors in clinical classification. Additionally, partial examinations were significantly more common at rehabilitation discharge than at admission, both in TSCI (cohort C vs. D) and NTSCI (cohort E vs. F) patients. This may be due to staffing limitations, time constraints, or clinical judgment, particularly if neurological function plateaued and reassessment was considered unnecessary. Furthermore, comparison of TSCI and NTSCI cohorts at rehabilitation revealed that the NTSCI group had significantly more examinations performed at rehabilitation admission (cohort C vs. E), but fewer were conducted at discharge (cohort D vs. F). For the TSCI group, the rehabilitation clinicians might rely on the exam done at acute discharge; while for the NTSCI group, attending rehabilitation at a RHSCIR facility might be the first point of specialized care where ISNCSCI is known as a best practice.

Analysis of missing ISNCSCI items in partial examinations revealed that motor scores were the most frequently completed, suggesting that clinicians may prioritize this part of the exam. Sensory scores (LT 53% and PP 55%) and rectal items (VAC 46% and DAP 50%) were most frequently omitted. Comparing TSCI and NTSCI cohorts at rehabilitation care, LT and PP were significantly more likely to be absent in the TSCI cohort compared to the NTSCI cohort, while VAC and DAP were more frequently absent in the NTSCI cohort compared to the TSCI cohort. This may be because NTSCI often results in more incomplete injuries.^17^ The sensory examination is subjective and can take a longer time to perform, particularly in patients with incomplete injuries. However, a sensory examination is essential in determining an accurate NLI in patients with SCI at the C1-4 or thoracic level. The rectal examination is an invasive procedure^4^ that requires careful explanation and patient consent before being performed. Due to its intimate nature, clinicians may be hesitant to perform or repeat this part of the examination unless well trained and fully aware of its clinical importance. Additionally, as patients undergo other rectal exams or procedures (e.g., methicillin-resistant *Staphylococcus aureus* [MRSA] rescreening, digital stimulation for bowel care, etc.), which cannot always be aligned, patients may perceive the examination as a repeated invasive procedure and may be reluctant to provide consent. Gaps in examinations at discharge may also reflect clinicians’ perceptions of the value of reassessment at this time, when the patient may be more neurologically stable.

Osunronbi and Sharma^6^ reported that VAC and DAP were missing or not tested in 51% of the charts. These tests are crucial for determining sacral sparing, distinguishing complete/incomplete injuries, and informing both the AIS classification and patient's neurological diagnosis and prognosis. Omission of sacral examination findings in motor incomplete injuries may occur if clinicians believe it does not affect classification or management. While an accurate NLI and AIS can often be obtained with a partial sensory examination, an accurate AIS is less likely if rectal examination scores are missing, especially when sensory testing at S4/5 is also absent. Studies have reported accuracy rates of 80% to 95% using substitute, more rostral, exam scores for rectal exam scores.^18^

Use of the ISNCSCI worksheet was high (78%) across all cohorts, suggesting that the structured worksheet aids thorough examination completion. Similarly, Schuld et al.^19^ demonstrated that the worksheet improved classification accuracy. Thus, as more Canadian hospitals adopt electronic records, standardized worksheets and AI-assisted tools^20^ could enhance support for examination completion and documentation. Regarding classification accuracy, full examinations demonstrated strong agreement between clinician-determined and algorithm-generated AIS and NLI (mismatches of 10% and 6%, respectively). In contrast, partial examinations had larger mismatches (28% for AIS, 19% for NLI), particularly in acute care. This indicates that partial examinations are less accurate and may lead to inaccurate prognosis and suboptimal care.

Across all cohorts, 26% had no documented examination. Upon reviewing the reasons for the lack of examination, most cases were attributed to unknown/other causes. However, factors such as concurrent TBI and sedation/intubation were noted, particularly in acute care (cohorts A and B). Other studies^21^-^23^ have discussed the challenges of performing the ISNCSCI examination in the acute setting, highlighting injury severity, patient stability, and time constraints. Lampart et al.^24^ found that 46% of individuals with SCI lacked ISNCSCI examinations during hospitalization, while Osunronbi and Sharma^6^ reported an overall completion rate of 39%. The higher completion rate within RHSCIR facilities may be attributed to the network's implementation efforts.

In cohort A (TSCI-acute-admission), individuals without an ISNCSCI examination had more severe injuries, including lower GCS, higher ISS, and more frequent use of endotracheal tubes or tracheostomies, compared to those with an examination. Other studies also reported that associated injuries, head trauma, intoxication, and pain can affect examination completion and accuracy.^6^,^7^,^23^ These gaps in examination performance can result in inaccurate neurological diagnoses and hinder monitoring of SCI recovery. As such, other methods are being explored, for example, using blood, cerebrospinal fluid, and imaging biomarkers to assess injury severity and predict neurological outcomes.^25^-^28^ These approaches may be helpful for patients with concurrent injuries that prevent the use of the ISNCSCI examination.

The expedited ISNCSCI (E-ISNCSCI_V1; https://asia-spinalinjury.org/expedited-isncsci-exam/) assessment provides a faster, standardized evaluation of NLI and AIS by omitting nonessential items (e.g., elements of sensory testing) and allowing optional inference of others (e.g., rectal components).^23^ The E-ISNCSCI_V1 should be performed by experienced examiners and is not a substitute for the full ISNCSCI. Given that the most common missing items in our study align with those omitted in E-ISNCSCI_V1, this assessment may support standardized partial exams. Since motor scores have significant clinical utility in monitoring neurological change, allow calculation of summed motor scores, and had the lowest rate of missing data, the E-ISNCSCI_V1 (which retains essential sensory items needed to determine and track changes in sensory levels) plus a full motor exam may serve as a practical alternative in early acute and rehabilitation care or when a full exam is not possible. This approach may also benefit research, as motor scores have been shown to be more responsive than sensory scores in detecting changes.^29^

This study shows that while current implementation efforts are helpful, more work is needed to improve ISNCSCI assessments for individuals with TSCI and NTSCI. Key steps may include collaborating with clinicians to establish a national standard that defines the appropriate ISNCSCI exam type (e.g., full or E-ISNCSCI_V1) at different care phases, enhancing worksheet use, ensuring integrated clinician training (e.g., ASIA's InSTeP course and hands-on workshops), and addressing modifiable factors like staffing shortages and time constraints. Additionally, integrating technology, such as the ISNCSCI algorithms,^10^,^30^-^32^ can support continuous learning and enhance classification. Standardizing protocols, incorporating assessments into electronic health records, and conducting regular audits will streamline the process. Importantly, clinicians need to recognize the value of the ISNCSCI as a crucial tool in the management of SCI, understanding its role not only in accurate classification but also in optimizing patient care and outcomes.

## Limitations

This study had a large SCI sample, however the NTSCI cohorts were smaller, and some facilities had fewer patients than others, which may have led to underrepresentation or bias in the comparisons of TSCI versus NTSCI. Future research using site stratification, multivariate, and sensitivity analyses could help reduce potential biases. As RHSCIR facilities receive access to ISNCSCI training and are encouraged to perform the first examination ≤72 hours, performance and accuracy may differ at nonparticipating facilities, which could impact the generalizability of our findings.

## Conclusion

This study evaluated ISNCSCI examination performance and classification accuracy in individuals with TSCI and NTSCI across RHSCIR-associated facilities in Canada, highlighting significant timepoint differences and barriers at the patient level and organizational level to examination completion. Future efforts should focus on addressing modifiable barriers to completion, integrating local clinical training, and determining the best standardized use of E-ISNCSCI_V1 across the network to improve research quality and patient outcomes.

## References

[1] Betz R, Biering-Sørensen F, Burns SP (2019). The 2019 revision of the International Standards for Neurological Classification of Spinal Cord Injury (ISNCSCI)— What's new?. Spinal Cord.

[2] Kirshblum S, Snider B, Rupp R, Read MS (2020). Updates of the International Standards for Neurologic Classification of Spinal Cord Injury: 2015 and 2019. Phys Med Rehabil Clin N Am.

[3] Kirshblum S, Schmidt Read M, Rupp R (2022). Classification challenges of the 2019 revised International Standards for Neurological Classification of Spinal Cord Injury (ISNCSCI). Spinal Cord.

[4] Parsons J, Plashkes T, Belanger L (2024). Toolkit for neurology assessment, version 3. A clinical guideline for the International Standards for Neurological Classification of Spinal Cord Injury (ISNCSCI). Praxis Spinal Cord Institute.

[5] Lena E, Baroncini I, Pavese C (2022). Reliability and validity of the international standards for neurological classification of spinal cord injury in patients with non-traumatic spinal cord lesions. Spinal Cord.

[6] Osunronbi T, Sharma H (2019). International Standards for Neurological Classification of Spinal Cord Injury: Factors influencing the frequency, completion and accuracy of documentation of neurology for patients with traumatic spinal cord injuries. Eur J Orthop Surg Traumatol.

[7] Snider BA, Eren F, Reeves RK, Rupp R, Kirshblum SC (2023). The International Standards for Neurological Classification of Spinal Cord Injury: Classification accuracy and challenges. Top Spinal Cord Inj Rehabil.

[8] Fallah N, Noonan VK, Waheed Z (2022). Development of a machine learning algorithm for predicting inhospital and 1-year mortality after traumatic spinal cord injury. Spine J.

[9] Praxis Spinal Cord Institute (2022). Rick Hansen Spinal Cord Injury Registry - A look at spinal cord injury in Canada in 2020.

[10] Walden K, Bélanger LM, Biering-Sørensen F (2016). Development and validation of a computerized algorithm for International Standards for Neurological Classification of Spinal Cord Injury (ISNCSCI). Spinal Cord.

[11] Parsons J, Plashkes T, Belanger L (2024). Toolkit for SCI neurology assessment. A clinical guideline for the International Standards for Neurological Classification of Spinal Cord Injury (ISNCSCI). Praxis Spinal Cord Institute.

[12] New PW, Simmonds F, Stevermuer T (2011). A population-based study comparing traumatic spinal cord injury and non-traumatic spinal cord injury using a national rehabilitation database. Spinal Cord.

[13] Lu Y, Shang Z, Zhang W (2024). Global incidence and characteristics of spinal cord injury since 2000–2021: A systematic review and meta-analysis. BMC Med.

[14] Alito A, Filardi V, Famà F (2021). Traumatic and non-traumatic spinal cord injury: Demographic characteristics, neurological and functional outcomes. A 7-year single centre experience. J Orthop.

[15] Scivoletto G, Farchi S, Laurenza L, Molinari M (2011). Traumatic and non-traumatic spinal cord lesions: An Italian comparison of neurological and functional outcomes. Spinal Cord.

[16] Rupp R, Biering-Sørensen F, Burns SP (2021). International Standards for Neurological Classification of Spinal Cord Injury: Revised 2019. Top Spinal Cord Inj Rehabil.

[17] Gedde MH, Lilleberg HS, Aßmus J, Gilhus NE, Rekand T (2019). Traumatic vs non-traumatic spinal cord injury: A comparison of primary rehabilitation outcomes and complications during hospitalization. J Spinal Cord Med.

[18] Zariffa J, Kramer JLK, Jones LAT, Lammertse DP, Curt A, Steeves JD (2012). Sacral sparing in SCI: beyond the S4-S5 and anorectal examination. Spine J.

[19] Schuld C, Franz S, Brüggemann K (2016). International standards for neurological classification of spinal cord injury: Impact of the revised worksheet (revision 02/13) on classification performance. J Spinal Cord Med.

[20] Bongurala AR, Save D, Virmani A, Kashyap R (2024). Transforming health care with artificial intelligence: Redefining medical documentation. Mayo Clin Proc Digit Heal.

[21] Pelletier-Roy R, Dionne A, Richard-Denis A, Briand M-M, Bourassa-Moreau É, Mac-Thiong J-M (2023). Validation of a new tool to detect and characterize spinal cord injury in the acute trauma patient: The Montreal acute classification of spinal cord injury. Glob Spine J.

[22] Battistuzzo CR, Smith K, Skeers P (2016). Early rapid neurological assessment for acute spinal cord injury trials. J Neurotrauma.

[23] Burns SP, Tansey KE (2020). The Expedited International Standards for Neurological Classification of Spinal Cord Injury (E-ISNCSCI). Spinal Cord.

[24] Lampart P, Gemperli A, Baumberger M (2018). Administration of assessment instruments during the first rehabilitation of patients with spinal cord injury: A retrospective chart analysis. Spinal Cord.

[25] Elizei SS, Kwon BK (2017). The translational importance of establishing biomarkers of human spinal cord injury. Neural Regen Res..

[26] Li J, Liu X, Wang J (2023). Identification of immunodiagnostic blood biomarkers associated with spinal cord injury severity. Front Immunol.

[27] Li J, Li J, Li X (2024;). Identification of coagulation diagnostic biomarkers related to the severity of spinal cord injury. Int Immunopharmacol.

[28] Morris S, Swift-LaPointe T, Yung A (2024). Advanced Magnetic resonance imaging biomarkers of the injured spinal cord: A comparative study of imaging and histology in human traumatic spinal cord injury. J Neurotrauma.

[29] Scivoletto G, Tamburella F, Laurenza L, Molinari M (2013). Distribution-based estimates of clinically significant changes in the International Standards for Neurological Classification of Spinal Cord Injury motor and sensory scores. Eur J Phys Rehabil Med.

[30] Walden K, Schuld C, Noonan VK, Rupp R (2023). Computer International Standards for Neurological Classification of Spinal Cord Injury (ISNCSCI) algorithms: A review. Spinal Cord.

[31] Schuld C, Franz S, Schweidler J (2022). Implementation of multilingual support of the European Multicenter Study about Spinal Cord Injury (EMSCI) ISNCSCI calculator. Spinal Cord.

[32] Schuld C, Wiese J, Hug A (2011). Computer implementation of the International Standards for Neurological Classification of Spinal Cord Injury for consistent and efficient derivation of its subscores including handling of data from not testable segments. J Neurotrauma.

